# Prognostic value of long non-coding RNA UCA1 in human solid tumors

**DOI:** 10.18632/oncotarget.11155

**Published:** 2016-08-09

**Authors:** Fang-teng Liu, Pei-qian Zhu, Hong-liang Luo, Yi Zhang, Cheng Qiu

**Affiliations:** ^1^ Department of General Surgery, the Second Affiliated Hospital of Nanchang University, Nanchang 330000, Jiangxi Province, P. R. China

**Keywords:** long non-coding RNA, UCA1, carcinoma, prognosis, meta-analysis

## Abstract

**Background:**

Numerous studies have shown that the expression of UCA1 was aberrantly upregulated in various cancer types. High expression of UCA1 was reported to be associated with unfavorable prognosis in cancer patients.

**Results:**

A total of 1240 patients from 15 articles were included. The results indicated that a significantly shorter OS was observed in patients with high expression level of UCA1 (HR = 1.71, 95% CI: 1.43–1.99), in the subgroup analysis, the association was also observed in patients with cancers of digestive system (HR = 2.12, 95% CI: 1.59–2.66). Statistical significance was also observed in subgroup meta-analysis stratified by the cancer type, cut-off value, analysis type and sample size. Furthermore, poorer DFS was observed in patients with high expression level of UCA1 (HR = 2.54; 95% CI: 1.09–4.00). Additionally, the pooled odds ratios (ORs) showed that increased UCA1 was also related to positive lymph node metastasis (OR = 2.98, 95% CI: 2.06–4.30), distant metastasis (OR = 3.14, 95% CI: 1.77–5.58) and poor clinical stage (OR = 2.76, 95% CI: 2.08–3.68).

**Materials and Methods:**

A comprehensive retrieval was conducted in multiple databases, including PubMed, Embase, Web of Science and CNKI. We collected relevant articles to explore the association between the expression levels of UCA1 and prognosis.

**Conclusions:**

High expression level of UCA1 was associated with poor clinical outcome. UCA1 could serve as a novel biomarker for prognosis and might be a potential predictive factor for clinicopathological characteristics in various cancers. Further studies should be performed to verify the clinical utility of UCA1 in human solid tumors.

## INTRODUCTION

Non-protein-coding genes have accounted for the vast majority of the human genome, while protein coding potential was observed in only ~2% of human genes. These abundant transcripts have been referred to as non-coding RNAs (ncRNAs). Long ncRNAs (lncRNAs) were one class of transcripts defined as transcribed RNA molecules greater than 200 nucleotides in length, without open reading frame of a significant length [1]. There is considerable evidence to suggest that lncRNAs were involved in a wide range of biological processes. They have emerged as critical factors in cancer progression [2–4]. Many studies have shown that lncRNAs played the role of oncogenes or cancer suppressors in tumor development [5–6]. Several lncRNAs, such as HOTAIR, MALAT1, and GAS5, were reported to be dys-regulated in cancer and were closely related to tumorigenesis, metastasis, and prognosis [7–9]. Hence, lncRNAs have opened up a new avenue towards to the researches on cancer initiation, progression, and metastasis. lncRNAs have showed their application potential in cancer diagnosis, prognosis and therapeutic target.

In recent years, a newly identified lncRNA, human urothelial carcinoma associated 1 (UCA1), has attracted much attention. This lncRNA was originally reported to play a role and its expression would be elevated in the oncogenesis of urinary bladder cancer [10]. UCA1 was found to promote tumorigenicity, with invasive potential in bladder cancer. In addition, the motility, invasion and drug resistance was enhanced by the ectopic expression of UCA1 in BLS-211 cells [11]. A number of other studies also revealed that UCA1 played a crucial role in carcinogenesis and that its expression would be elevated in a variety of malignancies [12–14]. Furthermore, the expression level of UCA1 was found to be associated with tumor clinicopathological features and patient prognosis. Therefore, in present study, relevant publications were collected and a meta-analysis was performed to investigate the relationship between UCA1 expression and clinical outcome. It aimed to further determine whether the over-expression of UCA1 could be applied as a potential biomarker for predicting the patient prognosis.

## RESULTS

### Study characteristics

**Figure 1:**
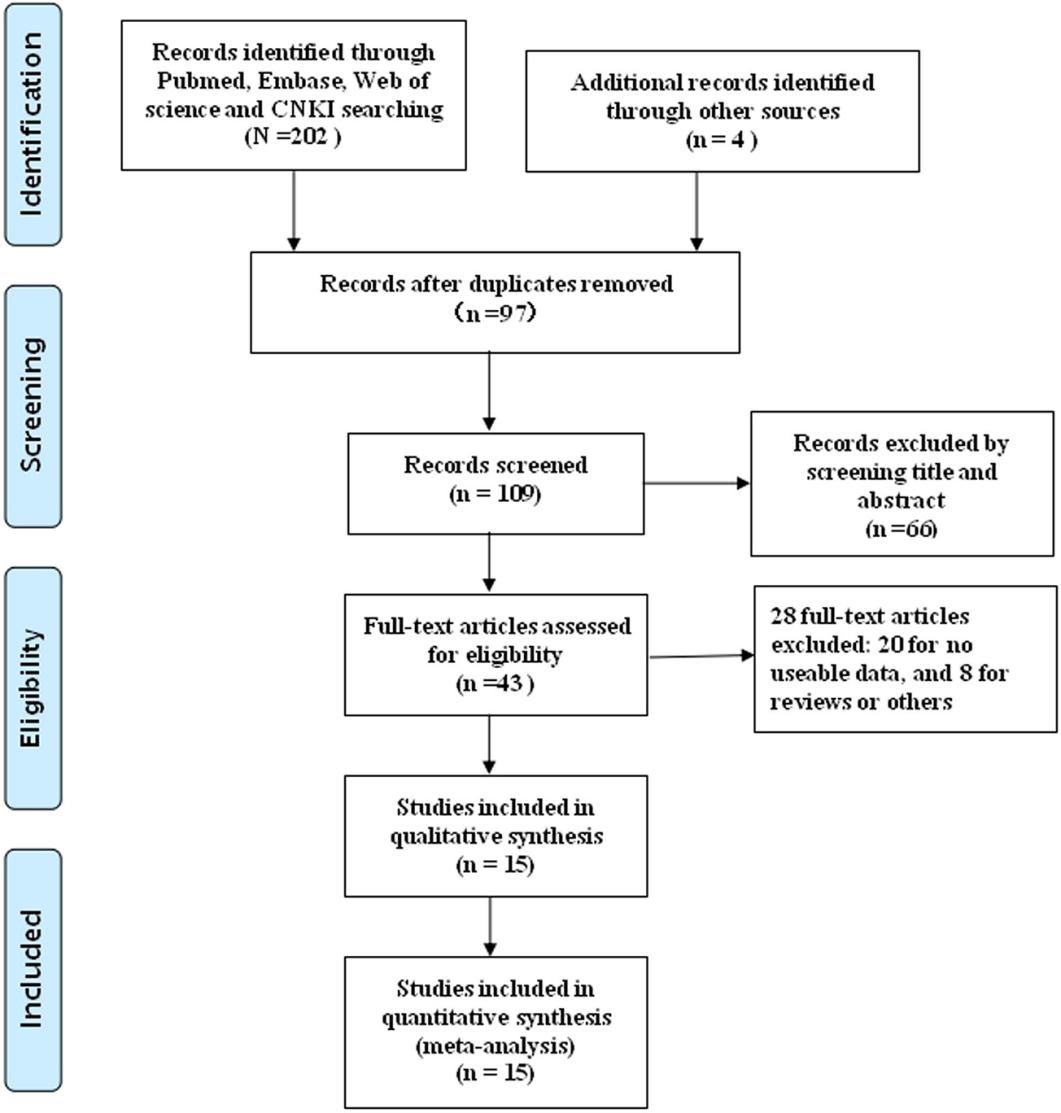
Flowchart presenting the steps of literature search and selection

The process of literature retrieval was shown in detail (Figure 1). A total of 15 eligible articles (16 studies) were ultimately identified [15–29]. A total of 1,240 cancer patients were included in present meta-analysis, and the mean patient sample size was 82.7 (ranging from 20 to 117). The included 16 studies were all conducted in China. Seven different solid tumor types were evaluated in our study, with five colorectal cancers (CRC), one esophageal squamous cell carcinomas (ESCC), one prostate cancer (PC), two hepatocellular carcinomas (HCC), two non-small cell lung cancers (NSCLC), two gastric cancers (GC) and two ovarian cancers (OC). All cancerous specimens were well preserved before RNA extraction. Diagnoses were all made based on pathology. The main characteristics were summarized (Table 1).

**Table 1:** Main characteristics of all included studies

First author	Year	Cancer type	Total number	Tumor stage(I/II/III/IV)	Follow-up(months)	Adjuvanttherapy beforesurgery	Criterion of high expression	Detectionmethod	Outcome measures	Multivariate analysis
Han Y[15]	2014	CRC	80	43/37(I–II/III–IV)	Mean 42.6	NR	mean expression	qRT-PCR	OS	no
Li JY[16]	2014	ESCC	90	39/51(I–II/III–IV)	median 43	None	mean expression	qRT-PCR	OS	yes
Cheng NN [29]	2015	NSCLC	52	NR	1–25	NR	the cut-off value	qRT-PCR	PFS	yes
Gao JF [17]	2015	GC	20	NR	1–40	None	NR	qRT-PCR	OS	yes
Tao K [18]	2015	CRC	80	44/36(I–II/III–IV)	Over 60	None	according to the fourth quartile of the expression level	qRT-PCR	OS	yes
Wang F [19]	2015	HCC	98	43/55(I–II/III–IV)	Over 60	None	median expression	qRT-PCR	OS	yes
Wang HM [20]	2015	NSCLC	60	28/32(I–II/III)	Over 60	None	median expression	qRT-PCR	OS	yes
Ni BB [21]	2015	CRC	54	35/19(I–II/III–IV)	Over 50	NR	median expression	qRT-PCR	OS	yes
Zheng Q [22]	2015	GC	112	39/73(I–II/III–IV)	Over 60	None	median expression	qRT-PCR	OS,DFS	yes
Na XY [28]	2015	PC	40	NR	Over 60	None	median expression	qRT-PCR	OS	no
Zhang L [23]	2016	OC	117	51/64(I–II/III–IV)	Median 22.0	None	median expression	qRT-PCR	OS	yes
Nie W [24]	2016	NSCLC	112	90/22(I–II/III)	Over 60	None	in relation to the Youdenindex	qRT-PCR	OS	yes
Yang YJ [25]	2016	OC	53	21/32(I–II/III–IV)	1–50	NR	median expression	qRT-PCR	OS	yes
Bian ZH-1 [26]	2016	CRC	90	37/53(I–II/III–IV)	Over 60	NR	median expression	qRT-PCR	OS	yes
Bian ZH-2 [26]	2016	CRC	105	80/39(I–II/III–IV)	Over 60	NR	median expression	qRT-PCR	OS	no
Shang C [27]	2016	HCC	77	NR	Over 60	None	NR	qRT-PCR	DFS	yes

Abbreviations: CRC: colorectal cancer; ESCC: esophageal squamous cell carcinoma; PC: prostate cancer; HCC: hepatocellular carcinoma; NSCLC: non-small cell lung cancer; GC: gastric cancer; OC: ovarian cancer; OS: overall survival; DFS: disease-free survival; PFS: progression free survival; qRT-PCR: quantitative real-time-polymerase chain reaction; NR: not reported.

### Increased UCA1 expression and OS

**Figure 2:**
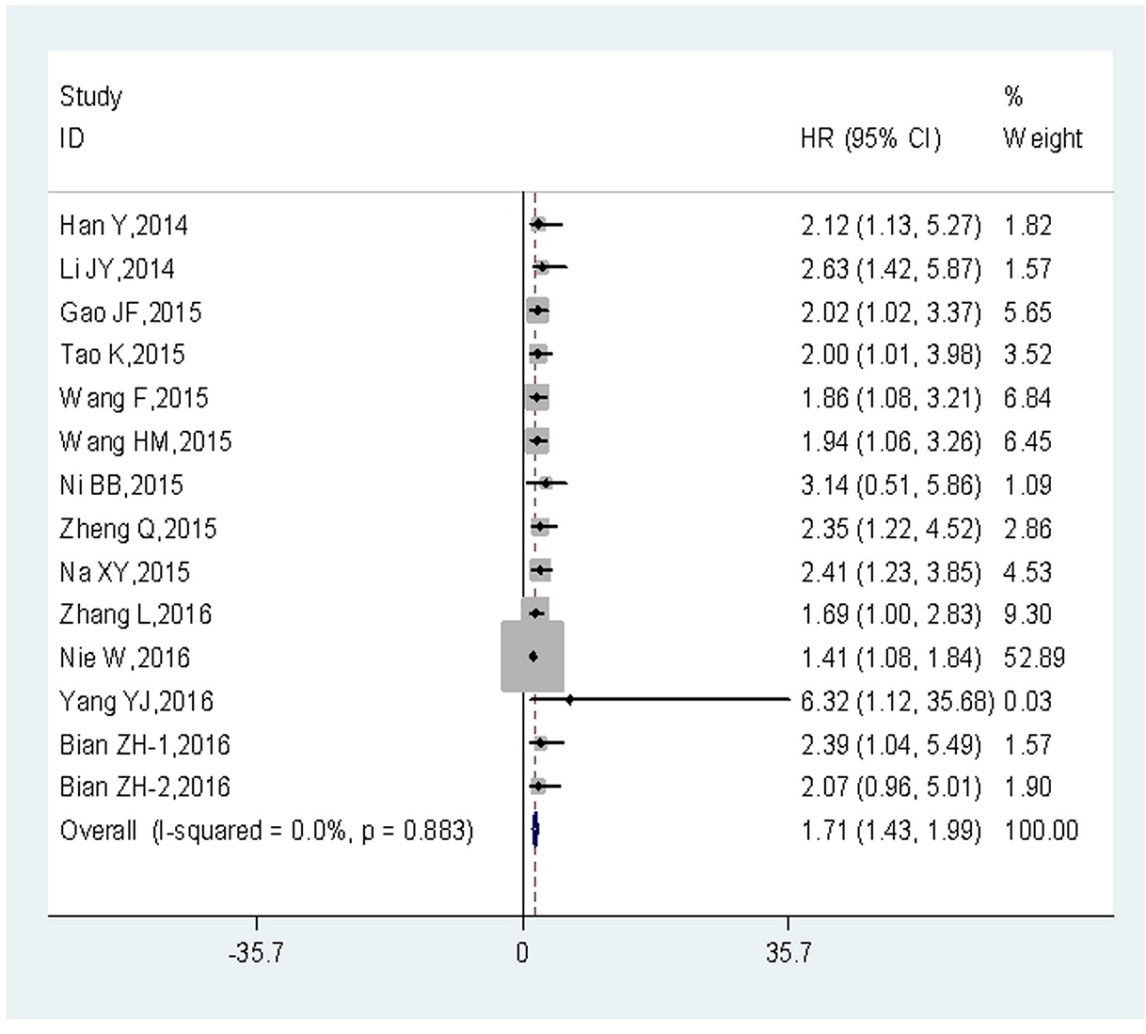
Forest plot of HR for the relationship between high UCA1 expression and OS

Among the 15 eligible articles, the OS according to UCA1 expression were reported in 13 articles (14 studies; 1,111 cancer patients). The fixed-effects model was adopted to estimate the pooled hazard ratios (HRs), as well as corresponding 95% confidence interval (CI). The result showed no heterogeneity across-studies (*I* ^2^ = 0%, *P_h_* = 0.883). The HRs, expressed as the high UCA1 expression group versus the low UCA1 expression group, was 1.71 (95% CI: 1.43–1.99, *P* = 0.000) (Figure 2). The result indicated that there was a significant difference in the OS between the two groups. A significantly shorter OS was observed in the patients with high UCA1 expression level, compared to those with low UCA1 expression level. Thus, it concluded that the expression of UCA1 at high levels was associated with poor OS.

**Figure 3:**
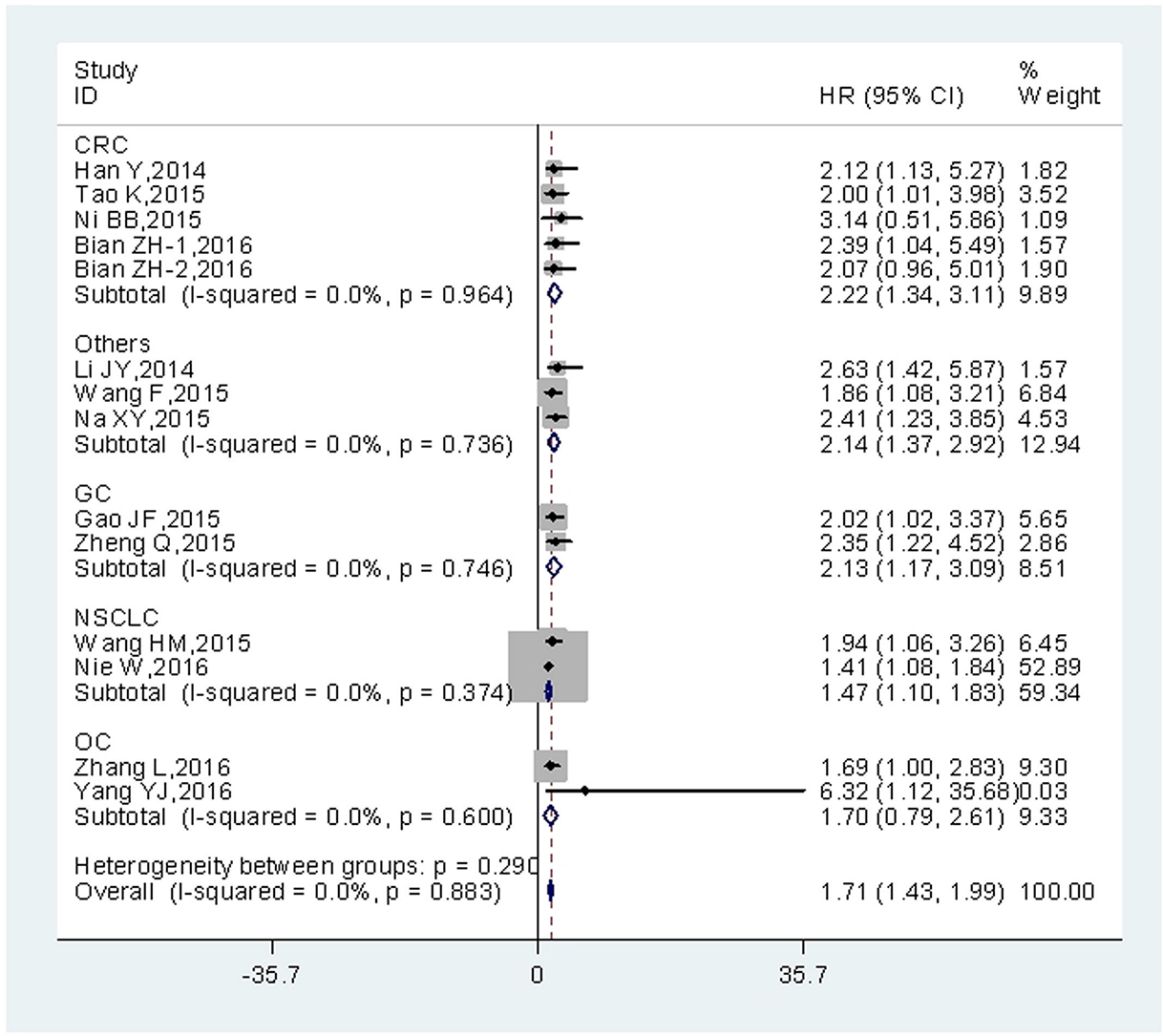
Forest plot of HR for the relationship between high UCA1 expression and OS in patients with various cancers

Additionally, pooled HRs for OS based on different types of tumor were shown (Figure 3). The negative effect of increased UCA1 expression on OS was demonstrated in patients with colorectal cancer (HR = 2.22; 95% CI = 1.34–3.11; *P* = 0.000), gastric cancer (HR= 2.13; 95% CI = 1.17–3.09; *P* = 0.000), non-small cell lung cancer (HR = 1.47; 95% CI = 1.10–1.83; *P* = 0.000) ovarian cancer (HR = 1.70; 95% CI = 0.79–2.61; *P* = 0.000) and other cancers (HR = 2.14; 95% CI = 1.37–2.92; *P* = 0.000). When all cancer types were roughly grouped into 2 categories (Digestive system cancers and others), a similar result was observed in digestive system cancers (HR = 2.12; 95% CI = 1.59–2.66; *P* = 0.000) (Table 2).

**Table 2:** Pooled HR for OS according to subgroup analysis

Categories	Studies (*n*)	Number of patients	Fixed-effects model		Heterogeneity	
			HR (95% CI) for OS	*P*-value	*I*^2^ (%)	*P_h_*
[1] OS	14	1111	1.71 (1.43–1.99)	0.000	0	0.883
[2] Cancer type						
1) Digestive system cancers	9	729	2.12 (1.59–2.66)	0.000	0	0.997
Others	5	382	1.55 (1.23–1.88)	0.000	0	0.553
2) CRC	5	409	2.22 (1.34–3.11)	0.000	0	0.964
GC	2	132	2.13 (1.17–3.09)	0.000	0	0.764
NSCLC	2	172	1.47 (1.10–1.83)	0.000	0	0.374
OC	2	170	1.70 (0.79–2.61)	0.000	0	0.600
Others	3	228	2.14 (1.37–2.92)	0.000	0	0.736
[3] Cutoff value						
Median	9	729	2.02 (1.55–2.49)	0.000	0	0.977
Mean	2	170	2.35 (0.84–3.87)	0.002	0	0.744
Others	3	212	1.50 (1.14–1.85)	0.000	0	0.497
[4] Analysis type						
Multivariate	11	886	1.66 (1.37–1.95)	0.000	0	0.828
Survival curves	3	225	2.27 (1.30–3.24)	0.000	0	0.951
[5] Sample size						
≥ 100	4	446	1.51 (1.17–1.85)	0.000	0	0.636
< 100	10	665	2.11 (1.63–2.60)	0.000	0	0.996

Moreover, for OS, the pooled HR values > 1 were consistently calculated in subgroup meta-analysis stratified by the cut-off value, analysis type and sample size, which was also statistically significant (Table 2, the Figures were presented in Supplementary Information).

### Increased UCA1 expression and DFS

**Figure 4:**
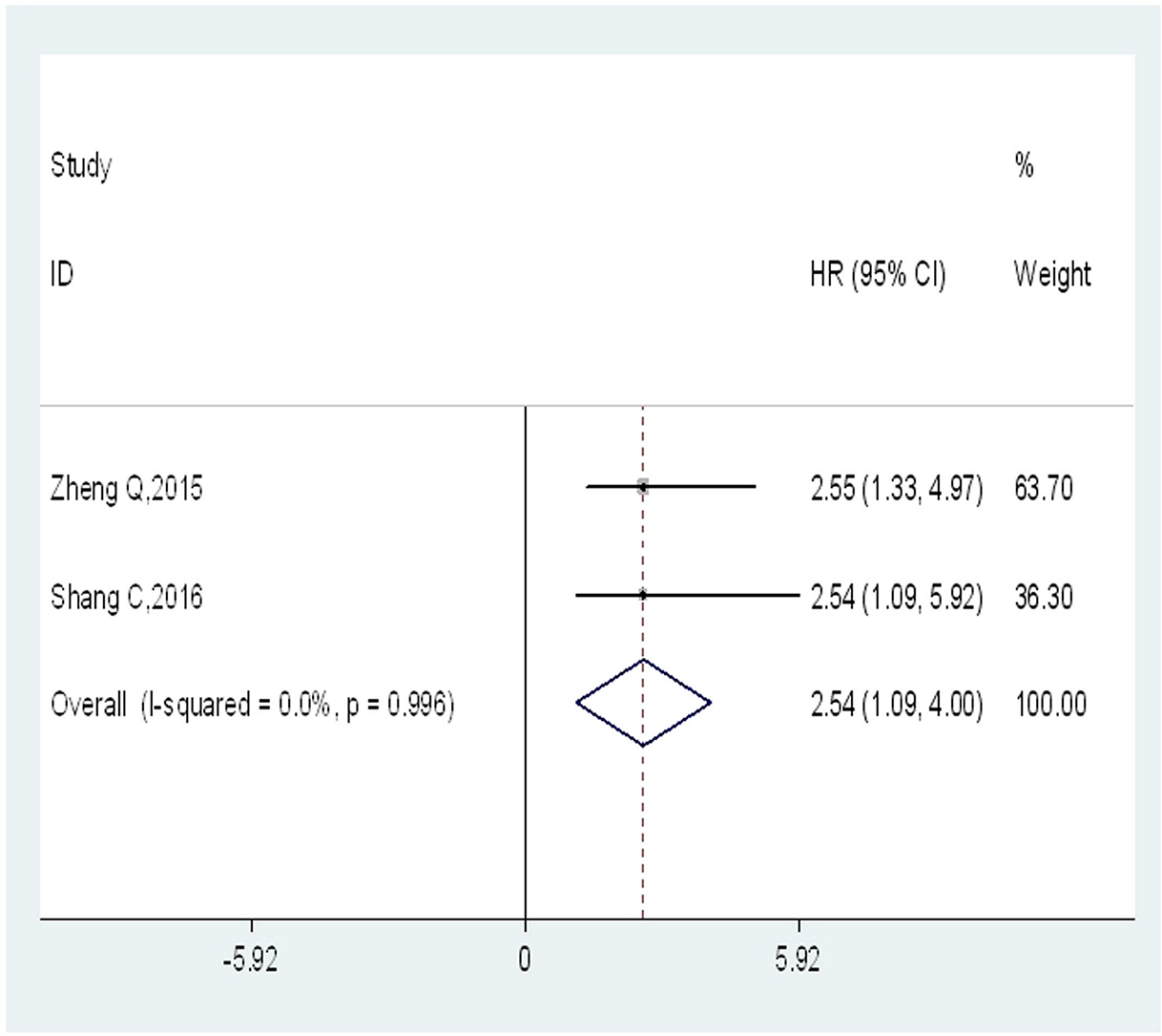
Forest plot of HR for the relationship between high UCA1 expression and DFS

Only two studies, comprising a total of 189 patients, provided appropriate data for DFS analysis. No severe statistical heterogeneity was observed across-studies (*I*^2^ = 0%; *P_h_* = 0.996), the fixed-effects model was applied to analyze the pooled hazard ratios (HRs) with corresponding 95% confidence interval (CI). The overall result indicated a significantly positive association between high expression level of UCA1 and poor DFS (HR = 2.54; 95% CI = 1.09–4.00; *P* = 0.001) (Figure 4).

### Associations between lncRNA-UCA1 expression and clinicopathological parameters

From the pooled results (Table 3), it found that increased UCA1 was significantly associated with lymph node metastasis (OR = 2.98, 95% CI: 2.06–4.30, *P* = 0.000), distant metastasis (OR = 3.14, 95% CI: 1.77–5.58, *P* = 0.000), and poor clinical stage (OR = 2.76, 95% CI: 2.08–3.68, *P* = 0.000). However, no significant correlation was observed between the increased UCA1 expression with the age, sex, tumor differentiation, lymphatic invasion and tumor size (All the figures were presented in Supplementary Information). Because of the insufficient data, we failed to detect the relationship between the over-expression of UCA1 and some other clinicopathological parameters.

**Table 3:** Meta-analysis results of the associations of increased UCA1 expression with clinicopathological parameters

Clinicopathological parameter	Studies (*n*)	Number of patients	OR (95% CI)	*P*-value	Heterogeneity		
					*I*^2^ (%)	*P_h_*	Model
Age (≥ 60 vs. < 60)	7	635	0.92 (0.67––1.28)	0.62	0	0.66	Fixed effects
Sex (Male vs. female)	9	776	0.77 (0.57–1.05)	0.09	0	0.45	Fixed effects
Lymphatic invasion (Present vs. Absent)	4	336	1.26 (0.80–1.99)	0.32	0	0.55	Fixed effects
Tumor size (≥ 5 vs. < 5)	5	460	1.41 (0.61–3.23)	0.42	78	0.001	Random effects
Tumor differentiation (Poorly/others vs. Well/moderately)	7	572	1.12 (0.78–1.59)	0.54	42	0.11	Fixed effects
Lymph node metastasis (Yes vs. No)	7	566	2.98 (2.06–4.30)	0.000	0	0.58	Fixed effects
Distant metastasis (Yes vs. No)	4	322	3.14 (1.77–5.58)	0.000	41	0.17	Fixed effects
TNM stage (III–IV vs. I–II)	10	884	2.76 (2.08–3.68)	0.000	6	0.39	Fixed effects

### Sensitivity analysis

For meta-analysis of the association between UCA1 expression level and OS, the sensitivity analysis was performed by removing each study in turn from the pooled analysis. It aimed to assess the influence of the removed data set on the overall HRs. The result was not significantly influenced after the exclusion of any study, indicating the robustness of the results.

### Publication bias

**Figure 5:**
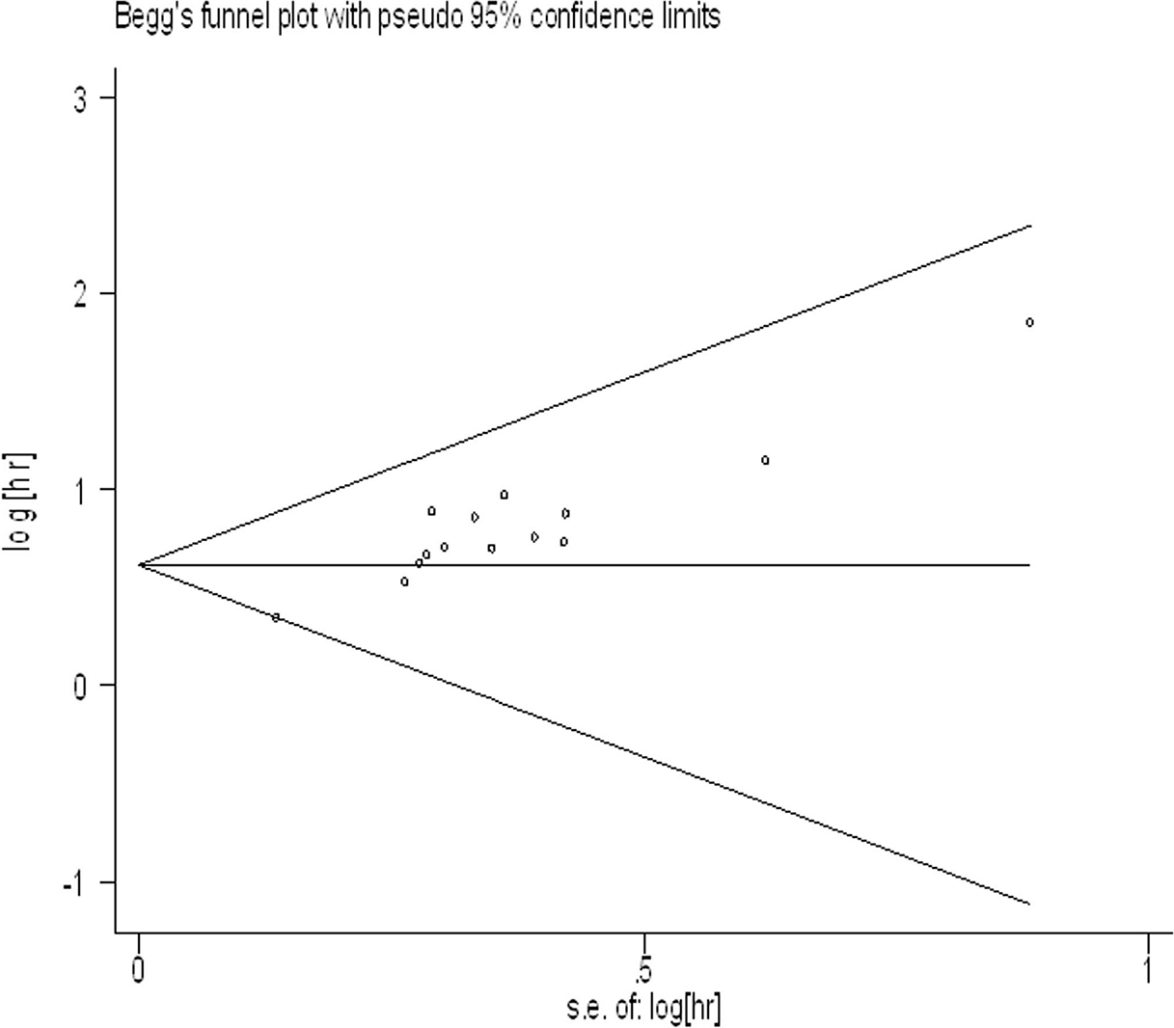
Funnel plot analysis of potential publication bias

For meta-analysis of the association between UCA1 expression levels and OS, the funnel plot was asymmetric (Figure 5), and the trim and fill method was also adopted to test for publication bias. The results showed no severe publication bias was observed between the included studies.

## DISCUSSION

UCA1 was a novel ncRNA gene, located in chromosome 19p13.12, which contained three exons and two introns. UCA1 was a long intergenic ncRNA that first discovered in bladder cancer in 2006 [10]. As an oncogenic lncRNA, it was reported to be abundant in many cancer cells and tumor tissues. UCA1 was involved in cancer progression, and its aberrant expression was associated with a broad range of cellular processes, including cell cycle distribution, apoptosis and proliferation [28, 30–31]. Importantly, UCA1 has been considered as a promising diagnostic marker and potential therapeutic target for human cancers [32–34]. Furthermore, some studies have shown that UCA1 was related to drug resistance in some malignancies, including ovarian cancer, bladder cancer and EGFR-mutant NSCLC, suggesting that UCA1 could be applied as a biomarker for monitoring the efficacy of chemotherapy [29, 35–36].

A growing number of studies have focused on the relationship between UCA1 and carcinoma, as well as interpreting its function in the progression of human cancer. However, its underlying molecular mechanism remained to be largely unclear. Numerous studies have shown that UCA1 made effects as an oncogenic lncRNA by inhibiting known tumor suppressors, such as p27 and miR-143 in breast cancer [37, 38] and BRG1 in bladder cancer [39]. It also acted as a molecular sponge for certain microRNAs (miRs), such as miR-193a-3p in NSCLC [24].

The mechanism on regulatory activity of UCA1 in cancer invasion and metastasis has been explored in several cancer types. In a study by Xue et al. [40], cell invasion of bladder cancer cells was promoted by UCA1 via the hsa-miR-145-ZEB1/2-FSCN1 pathway. Another study investigated on the function of UCA1 in hypoxic bladder cancer, revealing that UCA1 could function as a HIF-1α-targeted lncRNA to enhance bladder cancer cell invasion [41]. Chen et al. [42] found that the invasiveness of breast cancer cells could be promoted by the macrophage infiltration in the setting of up-regulated UCA1. Furthermore, a recent study confirmed a connection between UCA1 and miR-485-5p/MMP14 [25]. Regarding the role of UCA1 in HCC tumorigenesis, Wang et al. [19] found that UCA1 could facilitate HCC cell growth and metastasis through the inhibition of miR-216b and activation of the FGFR1/ERK signaling pathway. On the other hand, some studies have shown that aerobic glycolysis could be promoted by UCA1 in bladder cancer cells by targeting miR-16 and up-regulating hexokinase-2 [43–44], thus affecting their malignant potential.

Our meta-analysis provided evidence that high UCA1 expression was significantly correlated with a poor clinical prognosis in patients with various cancer types. Firstly, the combined results indicated that increased UCA1 expression was associated with a shorter OS in solid tumor patients. A shorter overall survival time was observed in the patients with high expression level of UCA1, compared to those with low UCA1 expression. It suggested that UCA1 could act as an independent prognostic factor for OS in cancer patients. In subgroup analysis for OS, UCA1 at high level in cancerous tissues may be a reliable prognostic marker specifically for digestive system cancers.

Secondly, this meta-analysis showed that patients with elevated UCA1 may suffer from a significantly poorer DFS. Only one study reported the PFS, thus we couldn't do further analysis. The result of both univariate and multivariate analysis indicated that the UCA1 expression level was an independent prognostic factor for PFS in patients with EGFR-TKI-sensitive NSCLC [29].

Thirdly, the clinicopathological significance of over-expressed UCA1 was also demonstrated in present meta-analysis. From the pooled results, we could find that increased UCA1 expression was positively correlated with advanced clinical stage, and cancer patients with high UCA1 expression may develop with an increased risk of LNM and DM.

However, there would be some limitations in our meta-analysis. For instance, the total sample size was relatively small, and patients included in the meta-analysis were all Asians from China. Additionally, publication bias may exist, despite the fact that no significant publication bias was observed based on the trim and fill method, as well as stable results revealed in sensitivity analysis. Finally, the cut-off definition for high UCA1 expression was not consistent. Therefore, larger-size, multi-center and higher-quality studies with a unified criterion for determining UCA1 expression are necessary to validate the results in this study.

## MATERIALS AND METHODS

### Literature search

For obtaining potentially eligible studies, comprehensive literature retrieval was conducted in various databases: PubMed, Web of Science, Embase, and CNKI, with a cut-off date of April 11, 2016. The keywords for the search were as follows: “urothelial cancer associated 1”, “UCA1”, “lncRNA UCA1”, “cancer”, “carcinoma”, “neoplasm” and “tumor”. In addition, other relevant articles were also manually viewed from the references lists.

### Inclusion and exclusion criteria

Inclusion criteria for the articles were as follows: (1) the role of lncRNA UCA1 in the development of human cancer was investigated, (2) associations of lncRNA UCA1 expression with prognosis or clinicopathological features were described, (3) the expression level of lncRNA UCA1 in primary cancerous tissue was determined by RT-qPCR and (4) patients were divided into high and low expression groups according to the expression level of lncRNA UCA1.

Exclusion criteria for the articles were as follows: (1) duplicate publications; (2) studies without valuable data; and (3) reviews, letters, case reports and expert opinions.

### Date extraction and quality assessment

The data and information from all included studies were independently extracted by two investigators (ZY and QC). The following information were collected from each study: first author name, publication year, the study country, cancer type, total patients number, tumor stage, follow-up period, outcome measures, the criteria for high UCA1 expression, determination method, HR and corresponding 95% CI. Besides, the data of clinicopathological parameters were also extracted from the eligible studies. For studies that provided both the results of univariate and multivariate analysis, only the latter was selected because of its increased precision on interpreting confounding factors. If a study reported only Kaplan-Meier curves, Engauge Digitizer version 4.1 was applied to extract the survival data. In the event of a disagreement, a consensus was reached by a third investigator (ZPQ). The Newcastle-Ottawa Scale (NOS) was applied to assess the quality of all included studies. The NOS scores ranged from 0 to 9, and the study with an NOS score ≥ 6 was considered to be of high quality. The quality of all studies included in this meta-analysis was varied from 4 to 9, with a mean value of 6.5.

### Statistical methods

The current meta-analysis was performed with RevMan5.3 software and Stata SE12.0. The heterogeneity between studies was determined with the Chi square-based *Q* test and *I* ^2^ statistics. A *P* value less than 0.05 for the *Q* test and *I* ^2^ value above 50% were considered to be significantly heterogeneous. The fixed effects model was applied for studies with no obvious heterogeneity (P_*h*_ > 0.05, *I* ^2^ < 50%); otherwise, the random effects model was adopted (*P_h_* ≤ 0.05, *I* ^2^ ≥ 50%). Potential publication bias was assessed with a funnel plot. The sensitivity analysis was also performed to assess the stability of the results. A *P* value less than 0.05 was considered statistically significant.

## SUPPLEMENTARY MATERIALS FIGURES


