# Supplementary material for: Prognostic value of long non-coding RNA UCA1 in human solid tumors
Source: Oncotarget. 2016 Aug 9;7(36):57991–8000. doi: 10.18632/oncotarget.11155 (PMC5295406; doi:10.18632/oncotarget.11155)
Supplement: Supplementary file 1 [file oncotarget-07-57991-s001.pdf]

Prognostic value of long non-coding RNA UCA1 in human solid tumors

Supplementary Materials

(1) Association between lncRNA-UCA1 expression and OS:

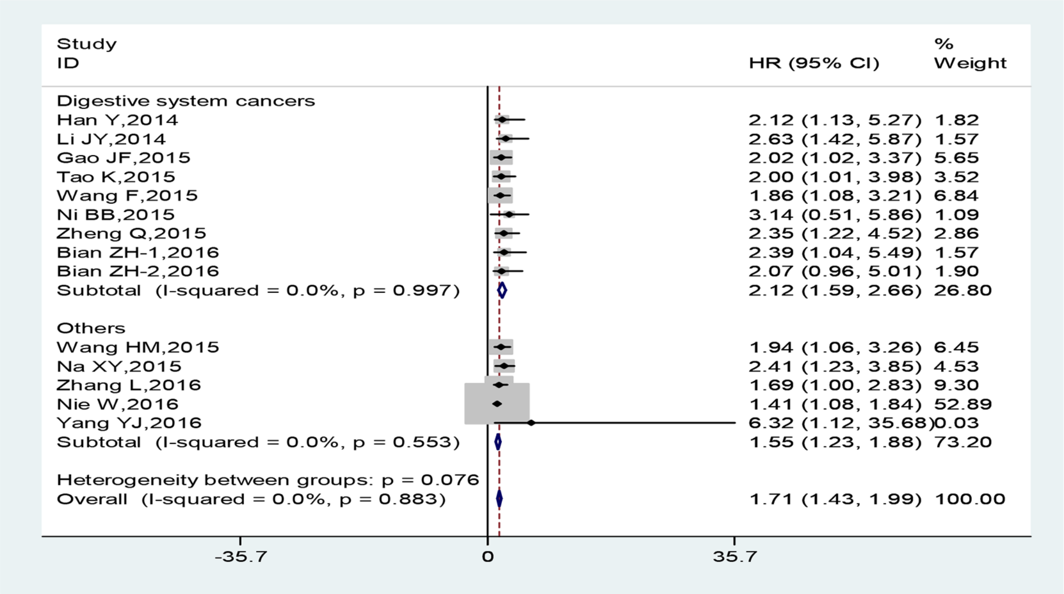

Supplementary Figure S1: Meta-analysis for the pooled HRs of OS stratified by the cancer type.

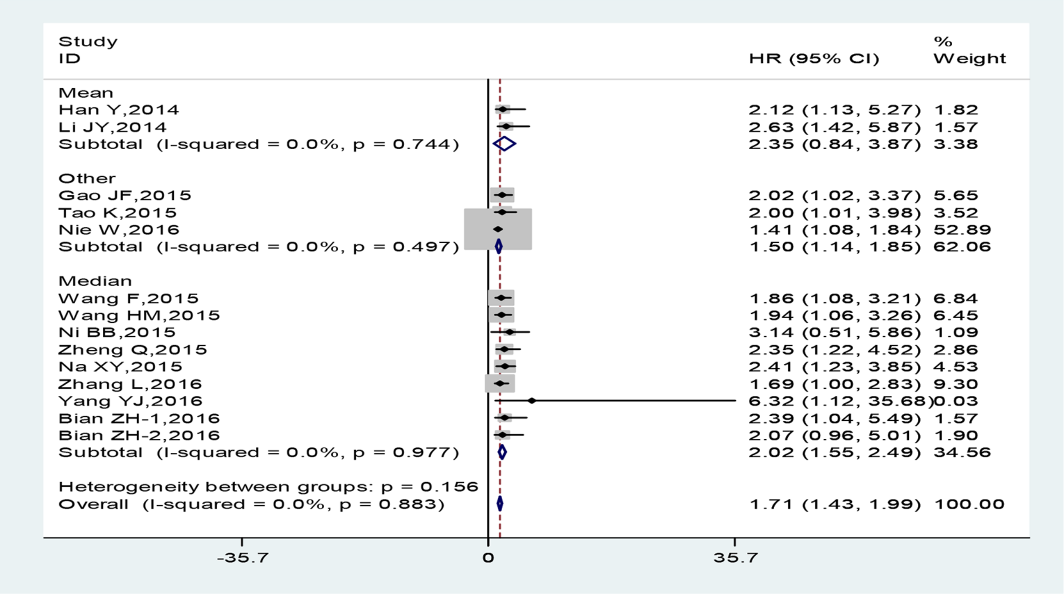

Supplementary Figure S2: Meta-analysis for the pooled HRs of OS stratified by the cutoff value.

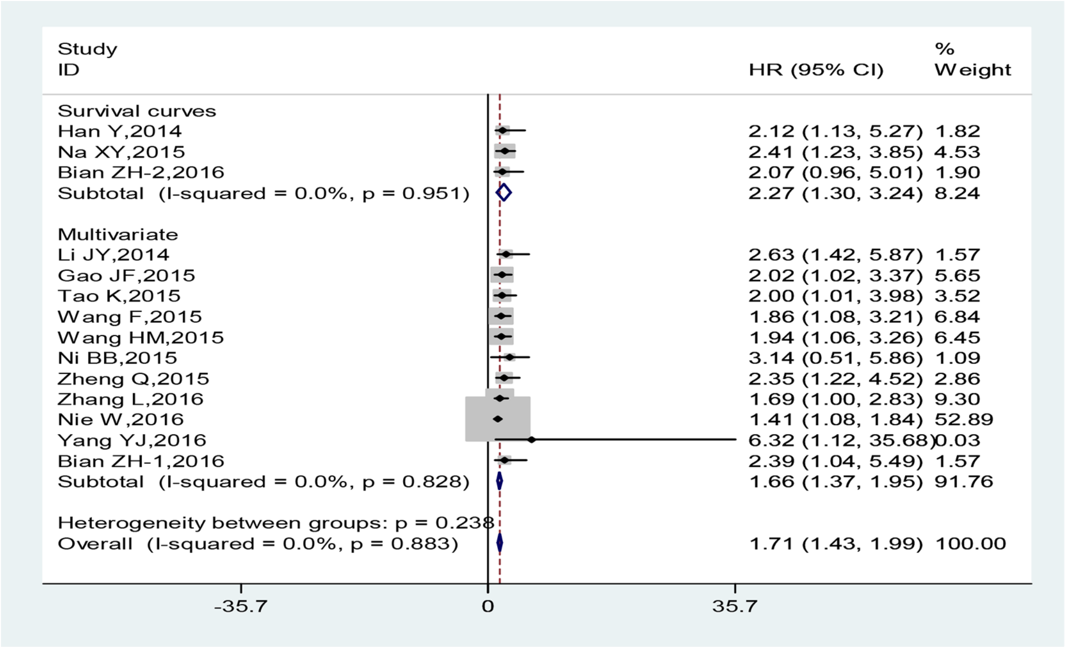

Supplementary Figure S3: Meta-analysis for the pooled HRs of OS stratified by the analysis type.

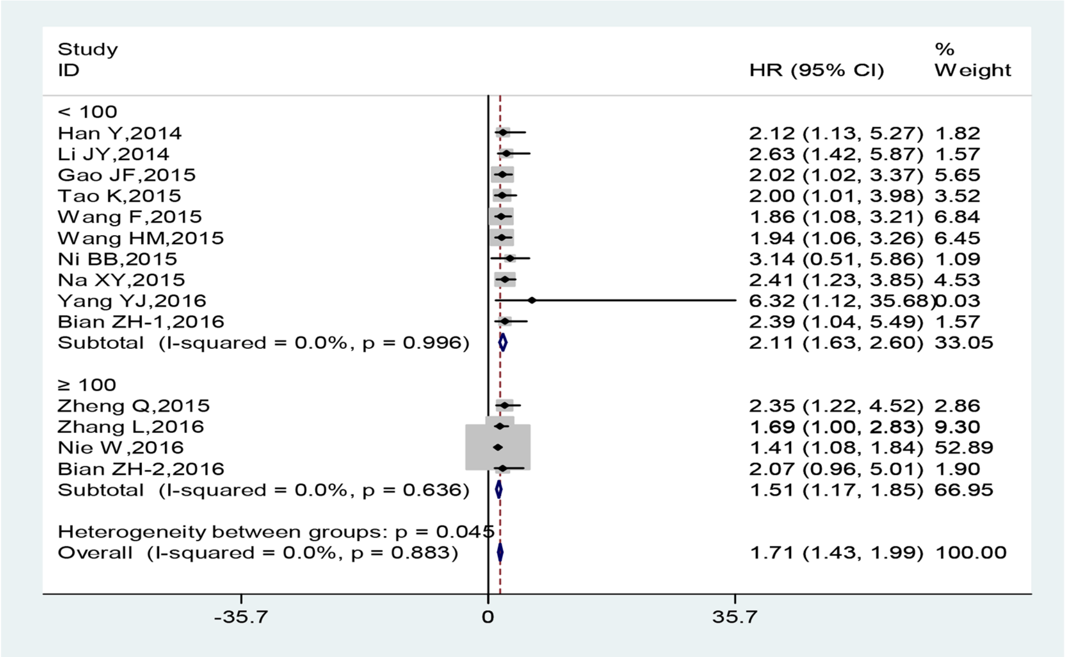

Supplementary Figure S4: Meta-analysis for the pooled HRs of OS stratified by the sample size.

(2) Association between lncRNA-UCA1 expression and clinicopathological parameters:

	≥ 60		< 60			Odds Ratio	
Study or Subgroup	Events	Total	Events	Total	Weight	M-H, Fixed, 95% CI	Year
Li JY, 2014	20	41	21	49	12.9%	1.27 [0.55, 2.92]	2014
Ni BB, 2015	18	35	9	19	7.5%	1.18 [0.38, 3.60]	2015
Zheng Q, 2015	37	77	19	35	17.9%	0.78 [0.35, 1.74]	2015
Wang HM, 2015	13	21	23	39	8.1%	1.13 [0.38, 3.35]	2015
Bian ZH, 2016	17	42	28	48	20.5%	0.49 [0.21, 1.13]	2016
Zhang L, 2016	36	74	23	43	19.7%	0.82 [0.39, 1.75]	2016
Nie W, 2016	26	70	13	42	13.5%	1.32 [0.58, 2.98]	2016
**Total (95% CI)**		**360**		**275**	**100.0%**	**0.92 [0.67, 1.28]**	
Total events	167		136				
Heterogeneity: Chi² = 4.11, df = 6 (P = 0.66); I² = 0%							
Test for overall effect: Z = 0.49 (P = 0.62)							

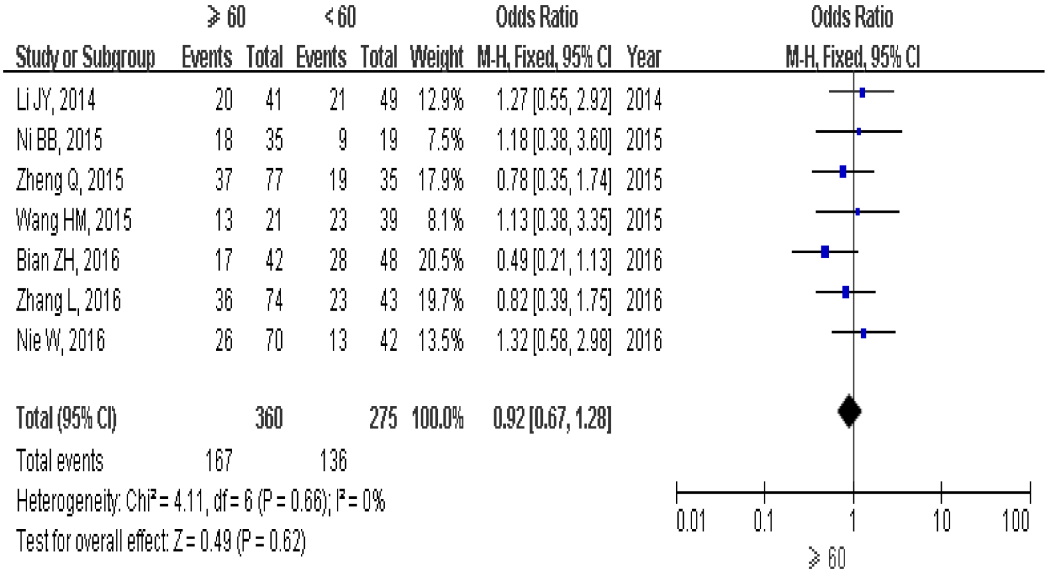

Supplementary Figure S5: The correlation between lncRNA-UCA1 expression and age.

	Male		Female			Odds Ratio	
Study or Subgroup	Events	Total	Events	Total	Weight	M-H, Fixed, 95% CI	Year
Han Y, 2014	16	39	21	41	12.6%	0.66 [0.27, 1.60]	2014
Li JY, 2014	24	50	17	40	10.3%	1.25 [0.54, 2.88]	2014
Ni BB, 2015	17	39	10	15	8.5%	0.39 [0.11, 1.34]	2015
Tao K, 2015	9	48	11	32	11.2%	0.44 [0.16, 1.23]	2015
Wang F, 2015	45	85	4	13	3.4%	2.53 [0.72, 8.86]	2015
Wang HM, 2015	22	37	14	23	7.3%	0.94 [0.33, 2.73]	2015
Zheng Q, 2015	30	64	26	48	16.5%	0.75 [0.35, 1.58]	2015
Bian ZH, 2016	23	49	22	41	13.3%	0.76 [0.33, 1.75]	2016
Nie W, 2016	20	67	19	45	16.7%	0.58 [0.26, 1.28]	2016
**Total (95% CI)**		**478**		**298**	**100.0%**	**0.77 [0.57, 1.05]**	
Total events	206		144				
Heterogeneity: Chi² = 7.80, df = 8 (P = 0.45); I² = 0%							
Test for overall effect: Z = 1.67 (P = 0.09)							

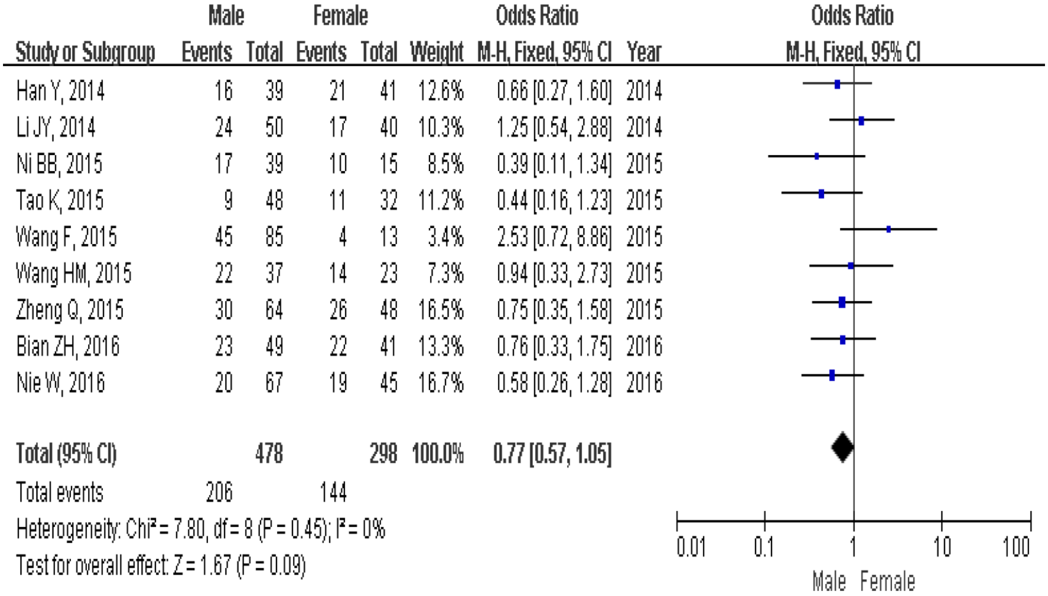

Supplementary Figure S6: The correlation between lncRNA-UCA1 expression and gender (male vs. female).

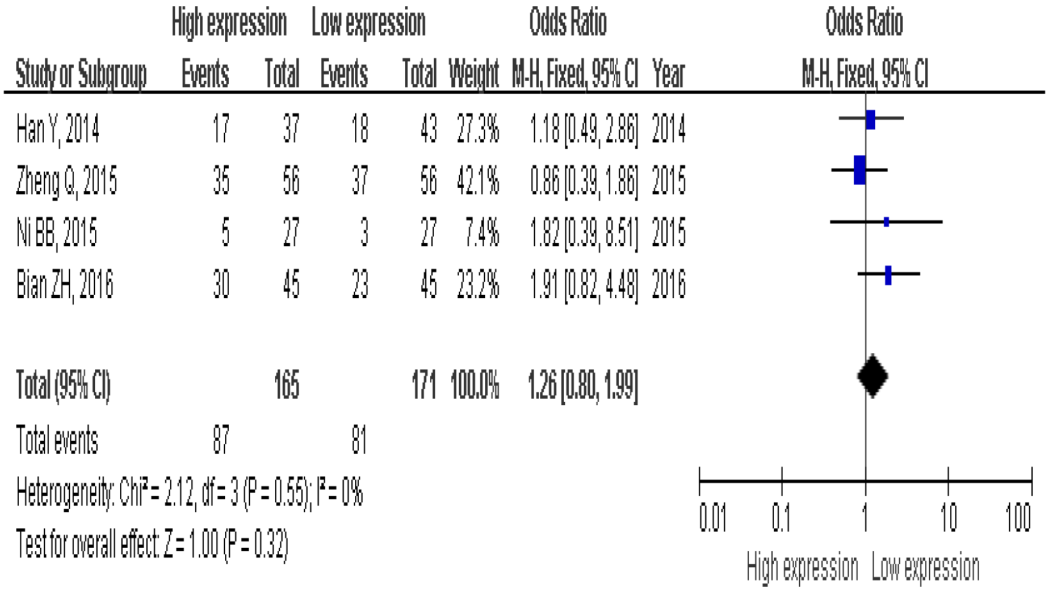

Supplementary Figure S7: The correlation between lncRNA-UCA1 expression and lymphatic invasion (present vs. absent).

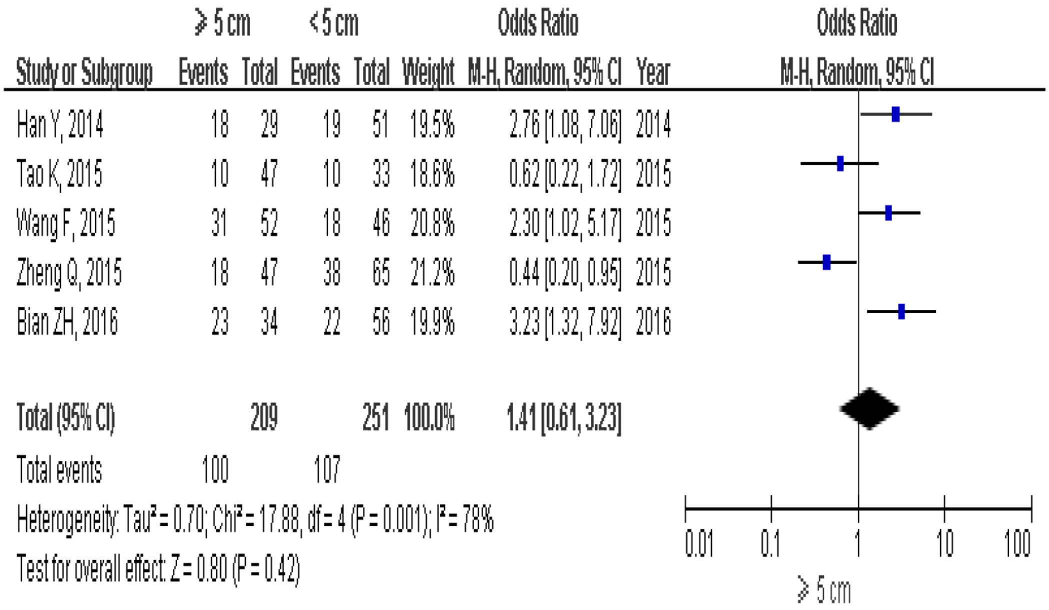

Supplementary Figure S8: The correlation between lncRNA-UCA1 expression and tumour size.

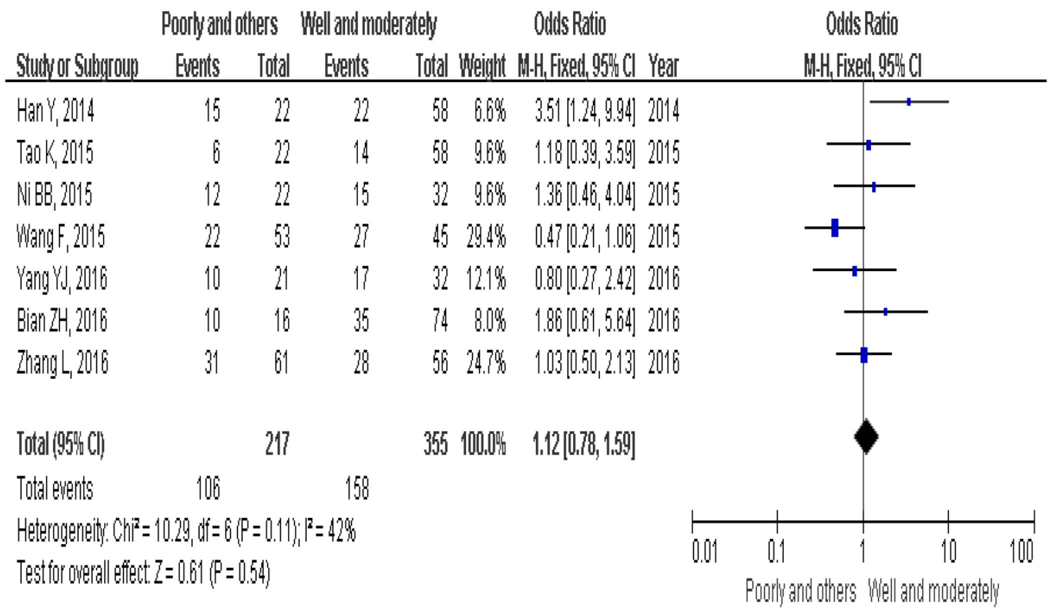

Supplementary Figure S9: The correlation between lncRNA-UCA1 expression and tumor differentiation (poorly/ others vs. well/moderately).

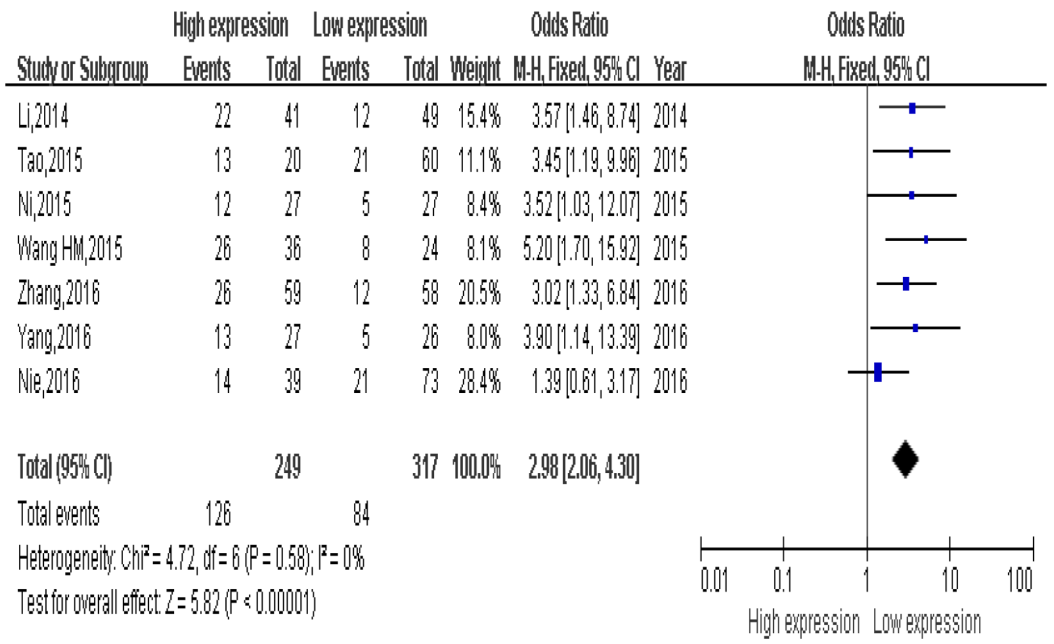

Supplementary Figure S10: The correlation between lncRNA-UCA1 expression and lymph node metastasis (yes vs. no).

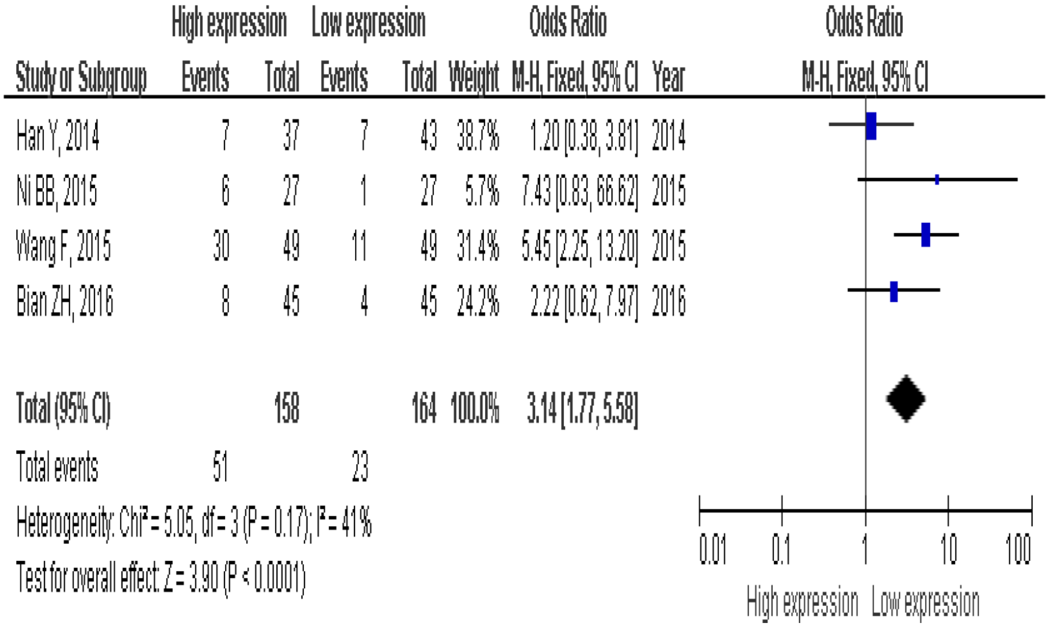

Supplementary Figure S11: The correlation between lncRNA-UCA1 expression and distant metastasis (yes vs. no).

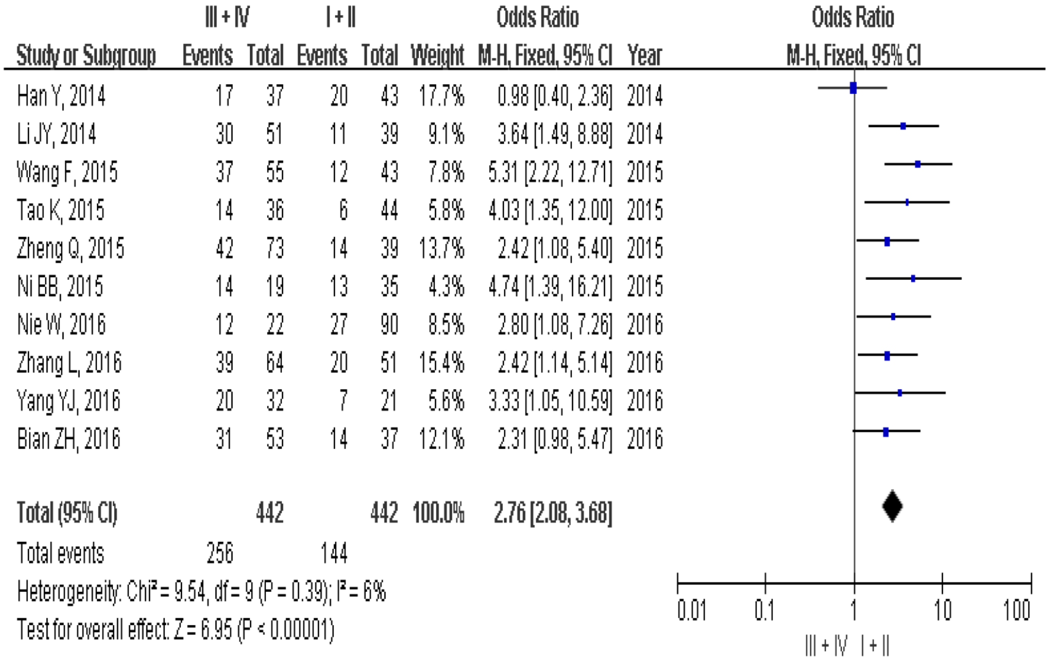

Supplementary Figure S12: The correlation between lncRNA-UCA1 expression and TNM stage (III–IV vs. I–II).
